# Variability among large language models in assessing CONSORT compliance of published randomized clinical trials

**DOI:** 10.1371/journal.pone.0358873

**Published:** 2026-09-22

**Authors:** Daniel Y. Tsybulnik, Justin J. Gillette, Thomas F. Heston

**Affiliations:** 1 Department of Medical Education and Clinical Sciences, Elson S. Floyd College of Medicine, Washington State University, Spokane, Washington, United States of America; 2 Department of Family Medicine, University of Washington School of Medicine, Seattle, Washington, United States of America; Islamic World Science & Technology Monitoring and Citation Institute (ISC), IRAN, ISLAMIC REPUBLIC OF

## Abstract

**Background:**

Peer review processes may inadequately assess compliance with established reporting guidelines such as the Consolidated Standards of Reporting Trials (CONSORT) criteria. Large language models (LLMs) demonstrate potential for systematic manuscript evaluation; however, how consistently different models assess adherence to CONSORT guidelines in published clinical trials remains unexplored.

**Methods:**

Twenty randomized controlled trials published in immunology journals between 2015 and 2016 were identified through PubMed. Three LLMs (ChatGPT-4o, Gemini 2.5 Flash, and Claude Sonnet 4.6) independently assessed compliance across 37 CONSORT 2010 subpoints. The primary endpoint was the difference between models in mean CONSORT compliance score. Secondary endpoints included inter-model agreement and the proportion of articles meeting a 90% compliance threshold. Statistical analysis employed repeated measures analysis of variance (ANOVA) with post-hoc pairwise comparisons (α = 0.05).

**Results:**

Mean CONSORT compliance rates were: ChatGPT-4o 80.8% (95% CI 75.8–85.8%), Gemini 2.5 Flash 64.4% (95% CI 58.0–70.8%), and Claude Sonnet 4.6 54.7% (95% CI 48.0–61.3%). Using a 90% compliance threshold as a quality benchmark, ChatGPT-4o identified 25% of papers (5/20) as meeting this standard, while Gemini 2.5 Flash and Claude Sonnet 4.6 each identified none (0/20) as meeting this standard. Repeated-measures ANOVA demonstrated significant differences between models (F(2,38) = 43.01, p < 0.001, partial η² = 0.694). All pairwise comparisons were statistically significant (ChatGPT-4o versus Gemini 2.5 Flash and ChatGPT-4o versus Claude Sonnet 4.6, both p < 0.001; Gemini 2.5 Flash versus Claude Sonnet 4.6, p = 0.014).

**Conclusions:**

LLMs varied substantially in their assessment of CONSORT compliance in published randomized trials, with a consistent ordering: ChatGPT-4o scored compliance highest, Gemini 2.5 Flash intermediate, and Claude Sonnet 4.6 lowest. Item-level agreement between models was only moderate, with no pairwise weighted kappa reaching the 0.61 substantial-agreement threshold. This inter-model variability indicates the need for standardized evaluation protocols before LLM-assisted manuscript screening is adopted.

## Introduction

Peer review serves as the primary quality control mechanism for scientific publications, yet studies consistently demonstrate substantial limitations in detecting methodological errors and reporting deficiencies [1]. The increasing strain on peer review processes, driven by rapid growth in manuscript submissions, has affected evaluation rigor across publishers [2]. When investigators intentionally introduced nine significant errors into randomized controlled trial manuscripts, peer reviewers detected an average of only three errors, with nearly 25% of reviewers identifying one error or fewer [3]. These systematic limitations extend to harm data reporting, where inconsistent analysis in randomized controlled trials fails to provide adequate information for clinical decision-making [4].

The Consolidated Standards of Reporting Trials (CONSORT) provides an evidence-based, minimum set of recommendations for reporting the methodology and results of randomized controlled trials to ensure clarity and transparency. First published in 1996 and subsequently updated in 2001, 2010, and 2025, CONSORT guidelines facilitate critical appraisal and enhance research reproducibility [5]. Despite widespread endorsement in high-impact medical journals, compliance remains suboptimal. Analysis of 463 abstracts from five leading journals found overall adherence of 67%, with individual journal rates ranging from 55% to 78% [6]. Similar shortfalls persist over time; a review of heart failure trials reported compliance between 60% and 70% across two decades [7].

Large language models (LLMs) have increasingly been utilized in scholarly processes, including literature reviews, manuscript preparation, and systematic error detection [8]. Advanced models, such as GPT-4, detect approximately 53% of intentionally inserted errors, approaching the performance of human peer reviewers [9]. When comparing LLM feedback to human reviewer comments on 3,096 manuscripts from Nature family journals, the overlap between GPT-4 and individual human reviewers (31%) closely matched inter-human reviewer agreement (29%) [10]. The practical significance of these capabilities was demonstrated when an AI model identified, within seconds, a ten-fold mathematical error in published flame-retardant research, a miscalculation that human reviewers had missed. This detection prompted the Black Spatula Project, an open-source initiative employing LLMs to identify overlooked errors in scientific literature [11]. LLMs have also demonstrated utility in automated paper screening for clinical reviews, extending their application to systematic literature evaluation [12].

How consistently different LLMs score the same trial against a structured reporting guideline such as CONSORT remains unexplored. If different models applied to the same manuscript produce systematically different compliance scores, the choice of model becomes a hidden determinant of any editorial or screening decision that relies on such scoring. This investigation compared three leading LLM platforms as raters of CONSORT compliance on a fixed set of 20 published randomized controlled trials, treating the trials as a common stimulus set and the models as the objects of study.

## Methods

### Study design and objectives

This investigation compared three LLMs as raters of CONSORT 2010 compliance on a fixed common set of 20 published randomized controlled trials. The trials functioned as a shared stimulus set; the models were the objects of study. The design does not estimate a population CONSORT compliance rate for any specialty or period, and sampling procedures relevant to prevalence estimation therefore do not apply. Trials were drawn only from publications preceding the April 2025 CONSORT update to ensure that all three models scored against the same 2010 criteria [5].

### Stimulus set

Randomized controlled trials were identified through a PubMed search using the search strategy: randomized controlled trial [Publication Type] AND immunology, filtered to full-text articles and restricted to a publication-year window of 2015–2016. The search was conducted in the fall of 2025. Fifty candidate articles were selected from the filtered result set using randomly generated record index numbers. Each candidate was screened against two criteria: that the article was a genuine randomized controlled trial, and that the publishing journal’s author guidelines required adherence to CONSORT reporting standards for randomized trials. Articles failing either check were excluded. Additional random index numbers were drawn until 50 qualifying articles had been identified. Twenty of these 50 qualifying articles were retained for LLM evaluation, with selection concluding once this sample size was judged sufficient for the planned repeated-measures analysis. The 20 retained trials were published between 2015 and 2016. Trials published after the 2025 CONSORT update were excluded to maintain methodological consistency throughout the evaluation period.

### Sample size determination

The evaluation used a fixed stimulus set of 20 randomized controlled trials, a size fixed in advance as adequate for the planned three-condition repeated-measures comparison of the models. The observed power for the within-subjects effect of model exceeded 0.999. A compact, uniformly scored stimulus set was preferred over a larger one that would not have changed the between-model comparison.

### CONSORT evaluation framework

The CONSORT 2010 checklist encompasses 25 primary items subdivided into 37 specific subpoints, each addressing essential elements of trial methodology and reporting [13]. This framework provides standardized criteria for evaluating the quality and transparency of randomized controlled trial reporting.

### Scoring methodology

Each article was evaluated across all 37 CONSORT subpoints using a three-level scoring system: complete fulfillment (1.0 point), partial fulfillment (0.5 point), or non-fulfillment (0 point). Subpoints assessed as not applicable to a given trial were scored as not fulfilled (0 point), and the denominator was fixed at 37 subpoints for every article to preserve comparability across trials. The LLMs independently determined fulfillment levels based on whether each CONSORT criterion was fully addressed in detail as specified in the CONSORT 2010 guidelines (1.0 point), partially addressed with incomplete detail or clarity (0.5 point), or not addressed (0 point). Overall compliance was calculated as the proportion of the maximum 37 points achieved across all subpoints.

### CONSORT compliance threshold

A 90% compliance threshold was selected as a clinically meaningful benchmark for high-quality reporting. While current adherence rates in leading medical journals range from 55% to 78%, with overall rates of approximately 67% [6], the CONSORT guidelines represent minimum standards for transparent trial reporting. For journals that endorse the CONSORT guidelines and frequently publish research that is cited in clinical practice, near-complete adherence to minimum reporting standards represents a reasonable quality expectation. This threshold serves as an aspirational benchmark against which current reporting practices can be evaluated.

### Large Language Model implementation

Three LLMs conducted parallel evaluations: ChatGPT-4o (OpenAI), Gemini 2.5 Flash (Google, free tier), and Claude Sonnet 4.6 (Anthropic, high-effort setting). All three models received the same standardized prompt with detailed instructions for criterion-by-criterion analysis and justification against each of the 37 CONSORT subpoints. Each model was accessed via its standard web interface, where generation parameters such as temperature are not user-configurable. Complete PDF manuscripts were uploaded to each platform, generating one independent evaluation per article per model. The complete standardized prompt provided to each LLM platform is available in S1 File. The list of evaluated articles with DOIs is provided in S2 File.

### Quality control and validation

All evaluated articles had previously undergone traditional peer review and been accepted for publication in immunology journals indexed in PubMed. Because no human gold-standard CONSORT assessment was performed, this study evaluates how the models differ in scoring compliance rather than their absolute accuracy against a validated reference standard.

### Statistical analysis

Statistical analyses were conducted following SAMPL (Statistical Analyses and Methods in the Published Literature) guidelines. A repeated measures analysis of variance (ANOVA) was performed with an α level of 0.05. The sphericity assumption underlying the within-subjects F test was evaluated using Mauchly’s test of sphericity. Sphericity was satisfied (W = 0.935, χ²(2) = 1.214, p = 0.545), so uncorrected, sphericity-assumed degrees of freedom were used and no epsilon correction was applied. The primary endpoint was the difference between models in mean CONSORT compliance score across the 37 subpoints. Secondary endpoints included inter-model agreement in assessments and the proportion of articles meeting a 90% compliance threshold. Descriptive statistics included means and 95% CIs. Post-hoc pairwise comparisons were performed using the Bonferroni correction for multiple testing. Effect sizes are reported as partial eta-squared (η²). Inter-model agreement was assessed using linear-weighted Cohen’s kappa for each pair of models and a two-way random-effects intraclass correlation coefficient (ICC; absolute agreement, single measures). Internal consistency across the three models was summarized with Cronbach’s alpha. Statistical analyses were conducted using IBM SPSS Statistics version 31 (IBM Corp., Armonk, NY, USA).

### Ethical considerations

This investigation utilized exclusively publicly available, peer-reviewed journal articles. No human subject research was performed.

## Results

### CONSORT compliance assessment

The three LLMs demonstrated substantial variability in CONSORT compliance assessment. Mean compliance rates were: ChatGPT-4o 80.8% (95% CI 75.8–85.8%), Gemini 2.5 Flash 64.4% (95% CI 58.0–70.8%), and Claude Sonnet 4.6 54.7% (95% CI 48.0–61.3%).

### Quality threshold analysis

**Fig 1:**
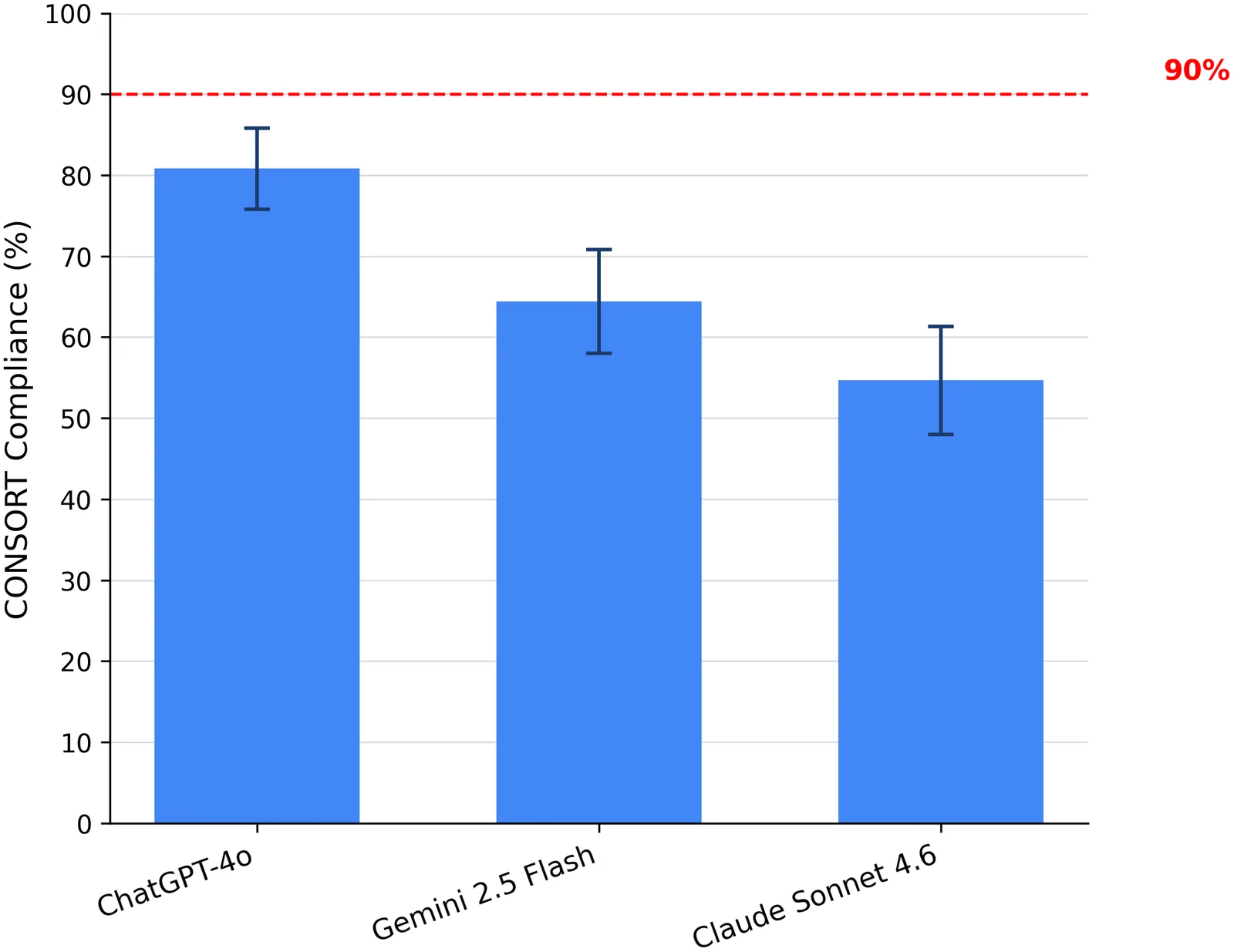
CONSORT compliance assessment by LLM platform. Mean CONSORT 2010 compliance rates for 20 randomized controlled trials evaluated by three LLM platforms. Error bars represent 95% CIs. The dashed line at 90% indicates the quality threshold benchmark. N = 20 articles per platform. Compliance was assessed using a three-level scoring system (0, 0.5, 1.0 point) across 37 CONSORT 2010 subpoints.

Using a 90% compliance threshold as a quality benchmark, ChatGPT-4o identified 5 articles (25%), while Gemini 2.5 Flash and Claude Sonnet 4.6 each identified no articles (0%) as meeting this standard. At least 75% of papers failed to meet the 90% threshold across all three models. These findings indicate substantial variation in how the models scored CONSORT reporting even among peer-reviewed publications (Fig 1).

### Statistical comparison between models

Mauchly’s test confirmed that the assumption of sphericity was met (W = 0.935, χ²(2) = 1.214, p = 0.545), so uncorrected degrees of freedom were used. Repeated-measures ANOVA demonstrated a statistically significant main effect of LLM type on CONSORT compliance scores (F(2,38) = 43.01, p < 0.001, partial η² = 0.694). This large effect size indicates substantial differences in how the three models assessed compliance.

Post-hoc pairwise comparisons using Bonferroni correction revealed statistically significant differences for all comparisons: ChatGPT-4o versus Gemini 2.5 Flash (mean difference 16.4 points, p < 0.001), ChatGPT-4o versus Claude Sonnet 4.6 (mean difference 26.1 points, p < 0.001), and Gemini 2.5 Flash versus Claude Sonnet 4.6 (mean difference 9.7 points, p = 0.014). ChatGPT-4o achieved the highest compliance scores, followed by Gemini 2.5 Flash and then Claude Sonnet 4.6.

### Inter-model agreement

Beyond differences in mean compliance scores, agreement between models on individual CONSORT subpoints was modest. Linear-weighted Cohen’s kappa was 0.308 (95% CI 0.259–0.358) for Claude Sonnet 4.6 versus ChatGPT-4o, 0.489 (95% CI 0.438–0.539) for Claude Sonnet 4.6 versus Gemini 2.5 Flash, and 0.368 (95% CI 0.306–0.429) for ChatGPT-4o versus Gemini 2.5 Flash, all p < 0.001. No pairwise value reached the 0.61 threshold conventionally regarded as substantial agreement, indicating that the three models frequently assigned different scores to the same CONSORT subpoint within the same trial. The two-way random-effects ICC for absolute agreement was 0.455 (95% CI 0.352–0.542) for single measures and 0.715 (95% CI 0.620–0.780) for the average of the three models (F(739, 1478) = 4.04, p < 0.001). Internal consistency across the three models was acceptable (Cronbach’s alpha = 0.752). Taken together, these values indicate that while the averaged rating across the three models is reasonably reliable, any single model is an unreliable substitute for another when scoring compliance at the item level.

Between-model disagreement was not uniform across the checklist but concentrated in specific subpoints (S3 Table). The largest divergence occurred for reporting of why the trial ended or was stopped (item 14b; range 0.70), the description of intervention similarity under blinding (item 11b; 0.55), losses and exclusions after randomization (item 13b; 0.53), ancillary analyses (item 18; 0.50), and generalizability (item 21; 0.48). The three models agreed almost completely on scientific background and objectives (items 2a and 2b; range 0.00), interpretation (item 22; 0.07), and the statistical methods for primary and secondary outcomes (item 12a; 0.10). Across the high-divergence subpoints, ChatGPT-4o assigned full compliance markedly more often than the other two models, which accounts for its higher overall mean. Disagreement therefore clustered in judgment-dependent reporting elements describing trial conduct and detailed results, rather than in structurally explicit items. The choice of model would consequently have its greatest effect on screening decisions for the subpoints where reporting quality is hardest to verify.

## Discussion

This investigation demonstrates that LLMs vary substantially in how they assess CONSORT compliance in published randomized controlled trials. The three models produced a consistent ordering: ChatGPT-4o scored compliance highest (80.8% mean), Gemini 2.5 Flash intermediate (64.4%), and Claude Sonnet 4.6 lowest (54.7%), and all pairwise differences were statistically significant. Even the highest-scoring model found that only 25% of papers met the 90% compliance threshold expected for high-quality clinical journals.

These findings occur within the broader context of well-documented peer review limitations [1]. Previous investigations demonstrate that peer reviewers detect only 3 of 9 intentionally inserted significant errors in randomized controlled trial manuscripts, with 25% of reviewers identifying only one error and 16% failing to detect any errors at all [3]. The increasing strain on peer review processes, driven by rapid growth in manuscript submissions, has reduced the time available for thorough evaluation of research quality and methodology [2]. Different peer review procedures demonstrate varying abilities to flag problematic publications, with systematic limitations across traditional editorial processes [14]. This investigation suggests that LLMs may serve as valuable complementary tools to address these systematic limitations in conventional editorial processes.

LLMs have demonstrated the ability to detect errors missed by peer review, such as the ten-fold dosage miscalculation in brominated diethyl ether research that prompted the Black Spatula Project, a grassroots initiative using LLMs to identify overlooked academic errors [11]. They have also been shown to detect commonly missed problems, including absent protocols, ambiguous ethics statements, and inappropriate citation practices [15].

Across all three models, at least 75% of papers failed to meet a 90% CONSORT compliance threshold. Inadequate CONSORT compliance compromises the ability of clinicians and researchers to appraise study methodology critically, assess risk of bias, and apply findings to patient care [16]. The potential for missed reporting errors to affect real-world clinical decisions underscores the importance of systematic quality assessment tools. Clinical research errors that escape detection can have substantial consequences. High-profile retractions for statistical problems illustrate this risk, including the 2023 retraction of a highly cited medication adherence study for misleading statistical reporting [17]. Among all reasons why a medical article may be deemed unfit for publication, the most common are plagiarism and data fabrication [18], but broader applications for error detection remain underutilized.

The design of this study identifies the magnitude of the differences between models but not their cause. Several explanations are plausible. Model architecture and the composition of training data differ across the three platforms, and models trained on different distributions of biomedical text may weight the same reporting elements differently [8]. The models also appear to apply different thresholds for what counts as adequate reporting: the divergence concentrated in judgment-dependent subpoints, such as why a trial was stopped, the description of intervention similarity under blinding, and losses after randomization, while structurally explicit subpoints, such as background and objectives, produced near-complete agreement. That pattern is consistent with the models differing in strictness when a criterion is partially addressed rather than differing in their ability to locate information in the text. Differences in how each model interpreted the shared prompt, and in the alignment and instruction-tuning procedures applied to each, may contribute as well. These explanations are plausible rather than established; the present design cannot separate them, and testing them would require systematic variation of prompt wording, model configuration, and criterion definitions. Because no human gold-standard assessment was available, the differences also cannot be attributed to one model being more accurate than another; they establish only that the models diverge systematically when scoring the same trials against the same criteria.

The relationship between LLM scoring and expert human assessment remains the central open question. When comparing LLM feedback to human reviewer comments on 3,096 papers from Nature family journals, the overlap between LLM and human assessments (31%) closely matched inter-human reviewer agreement (29%), with 82.4% of users finding GPT-4 feedback more helpful than some human reviewers [10]. However, a cross-sectional comparison of four LLMs against human reviewers across 22 manuscripts submitted to a surgical journal found that although the models were highly consistent across repeated evaluations (ICC = 0.88), they recommended rejection far less often than human reviewers, who rejected 68.2% of manuscripts compared with LLM rejection rates of 0 to 9.1% (p < 0.001) [19]. Agreement between the models and human recommendations in that study was moderate (weighted kappa 0.38 to 0.46), a range close to the item-level agreement observed among models in the present study.

Human CONSORT assessment is itself imperfect. When two trained reviewers independently scored published trials against the CONSORT checklist, they agreed completely on explicit items such as inclusion criteria, exclusion criteria, and the point estimate, but reached only moderate agreement on judgment-dependent items, with kappa values of 0.53 for allocation concealment and 0.54 for deviation from protocol [20]. That is the same pattern observed between models here. The relevant standard is therefore not perfect concordance but whether model-to-human agreement approaches the agreement observed between trained human assessors. The present study cannot address that question, because no human ratings of these 20 trials were obtained. The item-level agreement values reported here therefore describe only how interchangeable the models are with one another. Whether any of the three tracks expert human judgment, and which one tracks it most closely, requires a study in which the same trials are scored by both trained human CONSORT assessors and the models.

LLMs demonstrate particular strengths in linguistic editing, summarization, and systematic guideline evaluation, but may show limitations with statistics-heavy or nuanced medical content [8]. Previous research indicates that while LLMs can identify various types of errors comparable to human reviewers, they may exhibit weaknesses in evaluating complex statistical analyses and mathematical content [9]. Some studies have raised concerns about LLM limitations, including the generation of confident-sounding hallucinations with little relationship to actual paper content [21]. With certain prompting techniques or retrieval-augmented generation, newer LLM iterations can achieve lower hallucination rates on specific tasks compared to earlier models, though these improvements are often limited and do not eliminate the underlying issue [22].

These results extend previous investigations, demonstrating that GPT-4 detected 53% of intentionally inserted errors, comparable to the performance of human peer reviewers [9]. The current investigation focuses on systematic guideline compliance scoring rather than error detection. It shows that LLMs applying the same CONSORT criteria to the same trials produce systematically different compliance scores [23]. This application is distinct from previous work on linguistic editing, summarization, and basic error detection, and it addresses a gap in understanding how LLMs evaluate the quality of clinical trial reporting. Studies have demonstrated ChatGPT’s capability in identifying methodological flaws and providing insightful feedback on theoretical frameworks, with critical analyses aligning with human reviewers [24]. Recent work has also demonstrated LLM effectiveness in automated paper screening for clinical reviews, extending their utility beyond individual manuscript assessment to systematic literature evaluation [12].

Several limitations affect the interpretation of these findings. First, the trials were drawn from a single specialty, immunology, and from a narrow publication window, so the findings may not extend to other medical fields, other study designs, or other reporting contexts. The models may diverge to a different degree, or in a different order, when scoring trials whose reporting conventions differ from those in immunology. A single-specialty stimulus set nonetheless enabled a controlled comparison in which every model scored identical material, isolating between-model differences from variation in subject matter. Second, each model generated a single evaluation per article, so run-to-run variability within a model was not captured; the design nonetheless isolates differences between models under identical inputs. The partial scoring system (0/0.5/1.0) introduced subjective interpretation; conversely, it reflects real-world evaluation where criteria are often partially met. Third, no gold-standard human expert assessment was conducted, so this study characterizes how the models differ from one another rather than their absolute accuracy; establishing accuracy would require a validated human reference standard. Fourth, the stimulus set of 20 trials is small, and estimates derived from it are correspondingly imprecise. The sample supports comparison of the models against one another, but it does not support generalization of any single model’s absolute compliance score to the wider published literature. The set was nonetheless scored identically by all three models, and the large observed effect size indicates it was sufficient to detect the between-model differences under study. Fifth, LLMs may exhibit limitations in statistical content [8], although CONSORT predominantly assesses reporting transparency. Finally, CONSORT compliance represents only one quality dimension; however, this focused scope provides clear evidence for systematic differences between models in structured guideline evaluation.

Future research should prioritize human-AI agreement studies in which the same trials are scored independently by trained human CONSORT assessors and by multiple LLMs, reporting model-to-human agreement alongside human-to-human agreement so that model performance is judged against the reproducibility that human assessment actually achieves. Such studies would establish accuracy against a validated reference standard rather than the relative comparison reported here. Work should also focus on developing standardized LLM evaluation protocols for manuscript assessment and expanding evaluation to additional reporting guidelines such as the Preferred Reporting Items for Systematic Reviews and Meta-Analyses (PRISMA) and the Standards for Reporting of Diagnostic Accuracy Studies (STARD). Repeating the evaluation across multiple runs per model would quantify within-model variability alongside the between-model differences observed here. Investigation of prompt engineering techniques to optimize LLM performance and reduce inter-model variability represents another research priority. Traditional peer review has documented limitations in identifying research misconduct [14]. Whether LLMs can detect other forms of misconduct, including statistical fraud and citation manipulation, therefore warrants investigation. The continued evolution of LLM capabilities suggests expanding potential applications in scientific quality assessment [25].

## Conclusions

Three LLMs scoring the same 20 randomized controlled trials against the same 37 CONSORT subpoints produced significantly different compliance scores. Mean compliance ranged from 54.7% to 80.8%, with a consistent ordering of ChatGPT-4o highest, Gemini 2.5 Flash intermediate, and Claude Sonnet 4.6 lowest. Item-level agreement between models was only moderate, with no pairwise weighted kappa reaching the 0.61 substantial-agreement threshold. This degree of inter-model variability indicates that the choice of model materially affects the assessment, and that standardized evaluation protocols and validation against expert human review are needed before LLM-assisted compliance screening is adopted in editorial processes.

## Supporting information

S1 File Standardized CONSORT scoring prompt. The complete prompt provided identically to all three language models, specifying criterion-by-criterion evaluation against each of the 37 CONSORT 2010 subpoints.
(DOCX)

S2 File Evaluated articles with identifiers. List of the 20 randomized controlled trials assessed, including digital object identifiers.
(DOCX)

S3 Table Between-model divergence in CONSORT compliance scoring by subpoint. Mean compliance score for each of the 37 CONSORT 2010 subpoints, computed across the 20 randomized controlled trials for each model, with the range (highest minus lowest model mean) indicating disagreement; subpoints are ordered from greatest to least range.
(DOCX)
