# Supplementary material for: Variability among large language models in assessing CONSORT compliance of published randomized clinical trials
Source: PLoS One. 2026 Sep 22;21(9):e0358873. doi: 10.1371/journal.pone.0358873 (PMC13596804; doi:10.1371/journal.pone.0358873)
Supplement: Supplementary file 1 [file pone.0358873.s005.pdf]

Reviewer Comments to the Author

I appreciate the opportunity to review this revised manuscript. This study addresses an important and timely topic regarding the application of large language models (LLMs) in the evaluation of scientific reporting quality. The manuscript investigates an emerging issue by comparing three widely used LLM platforms in assessing CONSORT compliance among published randomized controlled trials. The research question is relevant, and the study provides useful insights into the variability of AI-based evaluation systems.

The manuscript has several strengths. The study design is clearly described, with the same set of randomized controlled trials evaluated by multiple LLMs using a standardized CONSORT-based framework. The authors have appropriately acknowledged that the study evaluates differences between models rather than absolute accuracy against a human reference standard. The statistical analysis is generally appropriate for the repeated-measures design, and the authors provide information regarding effect sizes, agreement measures, and limitations. The availability of the underlying data and analysis files also improves transparency and reproducibility.

I have several comments that may further strengthen the manuscript:

1. **Clarification of the interpretation of model performance**

The manuscript appropriately states that no human gold-standard assessment was available and therefore the study cannot determine which model was more accurate. However, some statements throughout the manuscript may still be interpreted by readers as implying superior performance of one model over another. I suggest maintaining careful wording throughout the Discussion and Conclusion to emphasize that the findings demonstrate variability in assessment behavior rather than differences in correctness.

2. **Further discussion of the implications of the scoring framework**

The authors used a three-level scoring approach (complete, partial, and non-fulfillment) across CONSORT subpoints. This approach is reasonable; however, some CONSORT items may involve interpretation rather than simple presence or absence of information. A more detailed discussion of how subjective interpretation may have contributed to model disagreement would improve the manuscript.

3. **Prompt standardization and model configuration**

The authors provide information that all models received the same standardized prompt and that complete prompt details are available in supplementary materials. However, additional discussion regarding potential effects of model-specific system instructions, interface differences, and non-user-configurable parameters (such as temperature settings) would be valuable, as these factors may influence reproducibility of LLM-based evaluations.

4. **Generalizability of findings**

The authors appropriately mention that the evaluated trials were limited to immunology journals. I suggest emphasizing this limitation more prominently in the abstract or conclusion, because model behavior may differ across specialties, reporting styles, and types of clinical research.

5. **Need for comparison with expert assessment**

A valuable direction for future research would be a direct comparison between LLM assessments and expert human CONSORT evaluations. Although the manuscript clearly acknowledges the absence of a human gold standard, highlighting this limitation earlier may help readers better understand the practical implications of the findings.

6. **Minor presentation issues**

The manuscript is generally well written and logically organized. Minor language polishing may improve readability in some sections, particularly where sentences are long and contain multiple concepts. Additionally, ensuring consistent terminology when referring to "compliance," "assessment," and "scoring" throughout the manuscript would improve clarity.

Overall, this is a relevant and well-conducted study that contributes to the growing discussion regarding the role of LLMs in scientific quality assessment. The manuscript would benefit from addressing the above points, mainly through clarification and refinement rather than major methodological changes.
