# Supplementary material for: Variability among large language models in assessing CONSORT compliance of published randomized clinical trials
Source: PLoS One. 2026 Sep 22;21(9):e0358873. doi: 10.1371/journal.pone.0358873 (PMC13596804; doi:10.1371/journal.pone.0358873)
Supplement: Supplementary file 3 [file pone.0358873.s002.docx]

**S2 File. Evaluated articles with identifiers.** List of the 20 randomized controlled trials assessed, including digital object identifiers.

1. De Santis O, Audran R, Pothin E, Warpelin-Decrausaz L, Vallotton L, Wuerzner G, et al. Safety and immunogenicity of a chimpanzee adenovirus-vectored Ebola vaccine in healthy adults: a randomised, double-blind, placebo-controlled, dose-finding, phase 1/2a study. The Lancet Infectious Diseases. 2016;16: 311–320. doi:10.1016/S1473-3099(15)00486-7
2. Haller MJ, Atkinson MA, Wasserfall CH, Brusko TM, Mathews CE, Hulme M, et al. Mobilization without immune depletion fails to restore immunological tolerance or preserve beta cell function in recent onset type 1 diabetes. Clinical and Experimental Immunology. 2016;183: 350–357. doi:10.1111/cei.12731
3. Fischer K, Bahlo J, Fink AM, Goede V, Herling CD, Cramer P, et al. Long-term remissions after FCR chemoimmunotherapy in previously untreated patients with CLL: updated results of the CLL8 trial. Blood. 2016;127: 208–215. doi:10.1182/blood-2015-06-651125
4. Beachler DC, Kreimer AR, Schiffman M, Herrero R, Wacholder S, Rodriguez AC, et al. Multisite HPV16/18 Vaccine Efficacy Against Cervical, Anal, and Oral HPV Infection. JNCIJ. 2016;108: djv302. doi:10.1093/jnci/djv302
5. Rapaport MH, Nierenberg AA, Schettler PJ, Kinkead B, Cardoos A, Walker R, et al. Inflammation as a predictive biomarker for response to omega-3 fatty acids in major depressive disorder: a proof-of-concept study. Mol Psychiatry. 2016;21: 71–79. doi:10.1038/mp.2015.22
6. Landin H, Mörk MJ, Larsson M, Waller KP. Vaccination against Staphylococcus aureus mastitis in two Swedish dairy herds. Acta Vet Scand. 2015;57: 81. doi:10.1186/s13028-015-0171-6
7. Raatz SK, Johnson LK, Picklo MJ. Consumption of Honey, Sucrose, and High-Fructose Corn Syrup Produces Similar Metabolic Effects in Glucose-Tolerant and -Intolerant Individuals ,. The Journal of Nutrition. 2015;145: 2265–2272. doi:10.3945/jn.115.218016
8. Genovese MC, Hsia E, Belkowski SM, Chien C, Masterson T, Thurmond RL, et al. Results from a Phase IIA Parallel Group Study of JNJ-40346527, an Oral CSF-1R Inhibitor, in Patients with Active Rheumatoid Arthritis despite Disease-modifying Antirheumatic Drug Therapy. J Rheumatol. 2015;42: 1752–1760. doi:10.3899/jrheum.141580
9. Ma JK, Drossard J, Lewis D, Altmann F, Boyle J, Christou P, et al. Regulatory approval and a first‐in‐human phase I clinical trial of a monoclonal antibody produced in transgenic tobacco plants. Plant Biotechnology Journal. 2015;13: 1106–1120. doi:10.1111/pbi.12416
10. Mily A, Rekha RS, Kamal SMM, Arifuzzaman ASM, Rahim Z, Khan L, et al. Significant Effects of Oral Phenylbutyrate and Vitamin D3 Adjunctive Therapy in Pulmonary Tuberculosis: A Randomized Controlled Trial. Mistry N, editor. PLoS ONE. 2015;10: e0138340. doi:10.1371/journal.pone.0138340
11. Bhatia A, K. Sharma R, Tewari S, C. Narula S. A randomized clinical trial of salivary substitute as an adjunct to scaling and root planing for management of periodontal inflammation in mouth breathing patients. Journal of Oral Science. 2015;57: 241–247. doi:10.2334/josnusd.57.241
12. Andrés C, Plana M, Guardo AC, Alvarez-Fernández C, Climent N, Gallart T, et al. HIV-1 Reservoir Dynamics after Vaccination and Antiretroviral Therapy Interruption Are Associated with Dendritic Cell Vaccine-Induced T Cell Responses. Silvestri G, editor. J Virol. 2015;89: 9189–9199. doi:10.1128/JVI.01062-15
13. Debrah AY, Specht S, Klarmann-Schulz U, Batsa L, Mand S, Marfo-Debrekyei Y, et al. Doxycycline Leads to Sterility and Enhanced Killing of Female Onchocerca volvulus Worms in an Area With Persistent Microfilaridermia After Repeated Ivermectin Treatment: A Randomized, Placebo-Controlled, Double-Blind Trial. Clin Infect Dis. 2015;61: 517–526. doi:10.1093/cid/civ363
14. Hunter DJ, Beavers DP, Eckstein F, Guermazi A, Loeser RF, Nicklas BJ, et al. The Intensive Diet and Exercise for Arthritis (IDEA) trial: 18-month radiographic and MRI outcomes. Osteoarthritis and Cartilage. 2015;23: 1090–1098. doi:10.1016/j.joca.2015.03.034
15. Nilsson C, Hejdeman B, Godoy-Ramirez K, Tecleab T, Scarlatti G, Bråve A, et al. HIV-DNA Given with or without Intradermal Electroporation Is Safe and Highly Immunogenic in Healthy Swedish HIV-1 DNA/MVA Vaccinees: A Phase I Randomized Trial. Lama JR, editor. PLoS ONE. 2015;10: e0131748. doi:10.1371/journal.pone.0131748
16. Lo MM, Mbao V, Sierra P, Thiongane Y, Diop M, Donadeu M, et al. Safety and immunogenicity of Onderstepoort Biological Products’ Rift Valley fever Clone 13 vaccine in sheep and goats under field conditions in Senegal. Onderstepoort J Vet Res. 2015;82: 6 pages. doi:10.4102/ojvr.v82i1.857
17. Fuchs CS, Azevedo S, Okusaka T, Van Laethem J-L, Lipton LR, Riess H, et al. A phase 3 randomized, double-blind, placebo-controlled trial of ganitumab or placebo in combination with gemcitabine as first-line therapy for metastatic adenocarcinoma of the pancreas: the GAMMA trial. Annals of Oncology. 2015;26: 921–927. doi:10.1093/annonc/mdv027
18. Joachim A, Nilsson C, Aboud S, Bakari M, Lyamuya EF, Robb ML, et al. Potent Functional Antibody Responses Elicited by HIV-I DNA Priming and Boosting with Heterologous HIV-1 Recombinant MVA in Healthy Tanzanian Adults. Reeves RK, editor. PLoS ONE. 2015;10: e0118486. doi:10.1371/journal.pone.0118486
19. Wheatley CM, Baker SE, Morgan MA, Martinez MG, Morgan WJ, Wong EC, et al. Effects of exercise intensity compared to albuterol in individuals with cystic fibrosis. Respiratory Medicine. 2015;109: 463–474. doi:10.1016/j.rmed.2014.12.002
20. Treven P, Mrak V, Bogovič Matijašić B, Horvat S, Rogelj I. Administration of probiotics Lactobacillus rhamnosus GG and Lactobacillus gasseri K7 during pregnancy and lactation changes mouse mesenteric lymph nodes and mammary gland microbiota. Journal of Dairy Science. 2015;98: 2114–2128. doi:10.3168/jds.2014-8519
