# Supplementary material for: Variability among large language models in assessing CONSORT compliance of published randomized clinical trials
Source: PLoS One. 2026 Sep 22;21(9):e0358873. doi: 10.1371/journal.pone.0358873 (PMC13596804; doi:10.1371/journal.pone.0358873)
Supplement: Supplementary file 4 [file pone.0358873.s003.docx]

**S3 Table**. **Between-model divergence in CONSORT compliance scoring by subpoint.**

| **CONSORT Subpoint** | **Claude** | **ChatGPT** | **Gemini** | **Range** |
| --- | --- | --- | --- | --- |
| 14b | .30 | 1.00 | .45 | .70 |
| 11b | .45 | 1.00 | .60 | .55 |
| 13b | .58 | 1.00 | .48 | .53 |
| 18 | .45 | .95 | .70 | .50 |
| 21 | .53 | 1.00 | .78 | .48 |
| 14a | .63 | 1.00 | .58 | .43 |
| 11a | .50 | .90 | .63 | .40 |
| 19 | .50 | .90 | .55 | .40 |
| 6b | .00 | .40 | .28 | .40 |
| 17a | .60 | .98 | .73 | .38 |
| 20 | .60 | .98 | .65 | .38 |
| 10 | .18 | .53 | .18 | .35 |
| 1b | .58 | .45 | .80 | .35 |
| 1a | .35 | .70 | .70 | .35 |
| 25 | .68 | 1.00 | .80 | .32 |
| 4a | .68 | 1.00 | .98 | .32 |
| 6a | .68 | 1.00 | .98 | .32 |
| 15 | .70 | 1.00 | .78 | .30 |
| 3b | .08 | .20 | .38 | .30 |
| 7b | .13 | .43 | .35 | .30 |
| 12b | .65 | .95 | .75 | .30 |
| 16 | .75 | 1.00 | .73 | .28 |
| 3a | .73 | 1.00 | .93 | .28 |
| 13a | .68 | .90 | .65 | .25 |
| 7a | .30 | .53 | .28 | .25 |
| 8b | .40 | .60 | .38 | .22 |
| 9 | .25 | .45 | .28 | .20 |
| 17b | .55 | .60 | .43 | .18 |
| 23 | .58 | .70 | .60 | .13 |
| 4b | .88 | 1.00 | .98 | .13 |
| 5 | .88 | 1.00 | 1.00 | .13 |
| 8a | .43 | .53 | .45 | .10 |
| 24 | .18 | .25 | .15 | .10 |
| 12a | .90 | 1.00 | 1.00 | .10 |
| 22 | .95 | 1.00 | .93 | .07 |
| 2a | 1.00 | 1.00 | 1.00 | .00 |
| 2b | 1.00 | 1.00 | 1.00 | .00 |

Mean compliance score for each of the 37 CONSORT 2010 subpoints, computed across the 20 randomized controlled trials for each language model (ChatGPT-4o, Gemini 2.5 Flash, Claude Sonnet 4.6), on a 0 to 1 scale where 0 indicates not addressed, 0.5 partially addressed, and 1.0 fully addressed. The range column is the difference between the highest and lowest model means for that subpoint; larger values indicate greater disagreement among models. Subpoints are ordered from greatest to least between-model range.
