# Supplementary material for: Variability among large language models in assessing CONSORT compliance of published randomized clinical trials
Source: PLoS One. 2026 Sep 22;21(9):e0358873. doi: 10.1371/journal.pone.0358873 (PMC13596804; doi:10.1371/journal.pone.0358873)
Supplement: Supplementary file 5 [file pone.0358873.s006.docx]

# Response to Reviewers

Manuscript PONE-D-25-55197

“Variability among large language models in assessing CONSORT compliance of published randomized clinical trials”

We thank the Academic Editor and both reviewers for their careful and constructive reviews. The comments identified several issues that materially improved the manuscript. In response, we made several substantial changes that affect the entire paper, along with numerous specific revisions detailed below.

First, we re-ran Claude Sonnet 4.6 using the same standardized, criterion-by-criterion prompt as the other two models, eliminating the prompt confound that both reviewers identified. All three models were then scored under identical instructions, inputs, and output requirements. Second, we rescoped the manuscript from a claim about model accuracy to one about inter-model scoring differences because no human gold-standard assessment was performed. Language throughout the Abstract, Results, Discussion, and Conclusions was revised accordingly, and we added formal inter-rater agreement analyses. The model reported in the original submission as Gemini 2.5 Pro was in fact Gemini 2.5 Flash; this identification has been corrected. In addition, during revision, the complete item-level scoring was re-verified by hand, coding discrepancies were corrected, and all statistics were recomputed on the resulting 740-observation dataset. Finally, the title was revised to reflect the rescoped focus, from the original wording referring to detecting compliance to "Variability among large language models in assessing CONSORT compliance of published randomized clinical trials," which states the study's actual finding and removes any implication of validated accuracy.

Because these changes recur across many individual comments, we describe them once here and reference them below where relevant. Point-by-point responses follow.

## Reviewer #1

**Comment 1.1** — The Introduction lacks a precise articulation of the methodological gap.

We agree. The Introduction previously summarized peer-review limitations and the CONSORT statement without providing a clear rationale for a comparative LLM evaluation. We revised the closing paragraph of the Introduction to state the gap directly: whether different LLMs, applying the same criteria to the same trials, produce consistent compliance assessments. The final sentence now frames the study as an evaluation of how three leading models differ in scoring CONSORT adherence, rather than a test of their accuracy.

**Comment 1.2** — Methods lack operational detail (prompts, temperature/generation parameters, input format, internal scoring criteria, self-evaluation, multiple runs, hallucination).

We agree and have addressed each element. The complete standardized prompt is provided in S1 File, and the Methods now point to it prominently. All three models were accessed through their standard web interfaces, in which generation parameters such as temperature are not user-configurable; we state this explicitly so readers understand the access conditions. Complete PDF manuscripts were uploaded to each platform. The three-level scoring instruction (met, partially met, not met) is specified in the prompt, and the mapping to 1, 0.5, and 0 is described in the Scoring Methodology section.

On self-evaluation and reproducibility, we agree this is a core constraint. The revised manuscript states that no human reference standard was used and that the study therefore characterizes differences among model scoring patterns rather than their correctness. One evaluation was generated per article per model; we now concede in the Limitations that run-to-run variability within a model was not captured, and we recommend repeated-run designs as future work.

**Comment 1.3** — The “first-20” sampling is not justified and may not be representative.

We agree. The Study Selection section has been rewritten to describe the actual sampling procedure: a PubMed search restricted to full-text randomized controlled trials in immunology; random selection of candidate articles by record index number; screening for genuine RCT design and journal-level CONSORT endorsement; and retention of 20 qualifying trials. This study does not estimate a population CONSORT compliance rate; each of the 20 articles was scored by all three models, so the comparison of interest is between models on a common, fixed set of trials rather than an estimate that depends on the sample being representative of the broader immunology literature. We nonetheless acknowledge single-specialty, single-period sampling as a limitation on how far the observed pattern of model divergence extends to other specialties or publication periods.

**Comment 1.4** — Results lack item-level granularity and formal agreement metrics (Cohen’s κ, ICC).

We agree and have added these analyses. The Results now report linear-weighted Cohen’s kappa for each model pair (Claude–ChatGPT 0.31, Claude–Gemini 0.49, ChatGPT–Gemini 0.37) and a two-way random-effects intraclass correlation coefficient (single measures 0.46; average measures 0.72), with Cronbach’s alpha 0.75. These analyses draw on the full 740-observation, item-level dataset, which has now been deposited in Zenodo. The agreement results show that no pairwise kappa values reach the substantial-agreement threshold of 0.61, which we highlight as a central finding. For item-level granularity, the Results now report the mean score of each model for every CONSORT subpoint and the between-model range for each, with full details in S3 Table. This analysis shows that disagreement concentrates in a subset of judgment-dependent subpoints, including why the trial ended or was stopped (item 14b), losses and exclusions (item 13b), and ancillary analyses (item 18), while the models agree almost completely on structurally explicit items such as background and objectives.

**Comment 1.5** — The 90% compliance threshold is arbitrary and insufficiently justified.

We agree the threshold is not a validated cutoff. We retained it as a descriptive benchmark because it offers an interpretable reference point, but we revised the text to present it explicitly as an aspirational benchmark rather than a validated quality standard, and we removed language implying that failing it demonstrates a reporting deficiency in an absolute sense. The Results now frame the threshold counts as a comparison among models rather than a verdict on the trials.

**Comment 1.6** — Discussion overstates implications and offers limited interpretation of why models differed; limitations are incomplete.

We agree. We removed the speculative characterization of one model as lenient and another as stringent, since that framing presumes a correct answer the design cannot establish. The Discussion now states plainly that the divergence establishes systematic differences between models, not the superiority of any one. On which domains drove the variability, the item-level analysis now identifies where disagreement concentrates: judgment-dependent reporting elements describing trial conduct and results detail, such as why the trial ended, losses and exclusions, blinding detail, ancillary analyses, and generalizability, with near-complete agreement on structurally explicit items. This provides the requested interpretation of where and on what kinds of items the models diverge. The Limitations section was expanded to address the absence of a human reference standard, the single-run design, single-specialty sampling, and the descriptive nature of the threshold, each paired with its mitigation.

We note one point respectfully. The revised manuscript avoids attributing the differences to prompt sensitivity, because a single-prompt design cannot support a prompt-sensitivity conclusion. We therefore did not add a prompt-sensitivity limitation, as it would imply a comparison the study did not perform; instead, we flag it as future work through an explicit factorial design.

**Comment 1.7** — Conclusions restate known literature rather than the study’s findings.

We agree. The Conclusions were rewritten to lead with the study’s own quantitative results — the significant differences among models, the range of mean compliance scores, the consistent ordering, and the moderate item-level agreement — followed by a single implication sentence on the need for standardized protocols and validation against expert review.

**Comment 1.8** — Several 2025 citations lack DOIs.

DOIs are now present for all references.

## Reviewer #2

We thank Reviewer #2 (Dr. Rasoul Zavaraqi) for a review that clarified precisely where our claims exceeded the scope of our design.

**Comment 2.1** — Absence of an expert human reference standard; “accuracy” is not validated by agreement with previously reported rates.

We agree entirely, and this comment drove the central revision. We removed the claim that agreement with published compliance rates validates model accuracy. The manuscript now states that no human gold-standard assessment was performed and that the design supports conclusions about differences among LLM scoring patterns, not about the correctness of those scores. The former Quality Control and Validation passage was rewritten to make this scope explicit.

**Comment 2.2** — Conclusions overstate the evidence; “accuracy,” “validated,” and “performance” should be replaced or qualified.

We agree. We revised this language throughout. The Abstract, Results, Discussion, and Conclusions now describe differences in inter-model scoring. Where “performance” implied correctness, it was replaced with “compliance scores” or “scoring.” The Abstract background was rephrased to ask how consistently models assess adherence, rather than how accurately. The Conclusions no longer claim validated accuracy.

**Comment 2.3** — Sampling strategy insufficiently described (search date, sorting, screening, inclusion/exclusion, journal list, exclusion reasons).

We agree. The Study Selection section now specifies the following elements: a PubMed search restricted to full-text randomized controlled trials in immunology; random selection of candidate articles by record index number; screening for RCT status and journal-level CONSORT endorsement; and retention of 20 qualifying trials. Selection within the filtered pool was random, addressing any concern that particular articles were chosen in a way that could favor one model's scoring over another's. Because all three models scored the same fixed set of trials, representativeness of that set affects only the generalizability of the model-divergence finding to other specialties or time periods, not the internal validity of the between-model comparison.

**Comment 2.4** — Prompt design differed across models; this is a major confound.

We agree, and we corrected it directly rather than by explanation. Claude Sonnet 4.6 was re-run under the identical standardized, criterion-by-criterion prompt used for ChatGPT-4o and Gemini 2.5 Flash. All three models now receive the same instructions, inputs, and output format, and all reported results derive from this standardized design. The single prompt is provided in S1 File. Because the study now uses one common prompt, we do not claim to test prompt complexity; we note an explicit factorial prompt design as a direction for future work, consistent with the reviewer’s suggestion.

We are grateful for the reviewers’ time and expertise. The revisions have produced a more transparent and accurately scoped manuscript, and we hope the revised version is now suitable for publication.

Thank you again for your consideration.
