# Supplementary material for: Variability among large language models in assessing CONSORT compliance of published randomized clinical trials
Source: PLoS One. 2026 Sep 22;21(9):e0358873. doi: 10.1371/journal.pone.0358873 (PMC13596804; doi:10.1371/journal.pone.0358873)
Supplement: Supplementary file 6 [file pone.0358873.s007.docx]

**RESPONSE TO REVIEWERS**

PONE-D-25-55197R1

Dear Dr. Danesh,

Thank you for your further review. All changes are marked in the tracked changes file.

**Journal requirements.** The reference list was verified. We corrected the formatting of references 2 and 25, updated reference 19 to the primary source, and added one reference (Moher 2001, now reference 20), shifting the subsequent numbering. Reference 17 is intentionally a retraction notice, cited and discussed as such.

**Reviewer #2.** We thank the reviewer for confirming the previous comments are resolved.

**Reviewer #3**

1. *Sample size and generalizability.* Both points were identified as limitations in the previous version and are now stated more explicitly in the Discussion limitations: a single specialty and narrow publication window may not extend to other fields, designs, or reporting contexts, and 20 trials support comparison between models but not generalization of any model's absolute score. The design remains matched to the question asked. Every model scored identical material, and the effect was large enough to resolve all three pairwise differences at n = 20.
2. *ANOVA assumptions.* We tested sphericity at the time of analysis and found it satisfied (Mauchly's W = 0.935, chi-square = 1.214 with 2 degrees of freedom, p = 0.545), so we used uncorrected degrees of freedom; the output was already in the deposited SPSS file but not reported in the text; it is now stated in the Statistical Analysis subsection and the Results.
3. *Why the models differ.* The Discussion now offers four candidate explanations: architecture, training data, strictness when a criterion is partially met, and prompt interpretation. These are anchored in the item-level results already reported, where divergence concentrated in judgment-dependent subpoints while structurally explicit subpoints showed near-complete agreement. The text states that the design cannot separate the four.
4. *Human reviewer comparison.* The previous version noted the absence of a human reference standard, and we maintain that model-to-model comparison answers a prerequisite question: if models disagree on the same text, at most one of them can track any fixed standard. The Discussion now grounds this in evidence: two trained reviewers scoring published trials against the CONSORT checklist agreed completely on explicit items but reached kappa of only 0.53 to 0.54 on judgment-dependent ones (Moher 2001), the same pattern seen between models here. Model-to-human agreement should therefore be judged against human-to-human agreement rather than treated as accuracy against truth. The recommendation for human-AI agreement studies now opens the future research paragraph.
5. *Language.* Seven long sentences were split, and one Discussion paragraph was divided. No numbers changed.

Sincerely,

Thomas F Heston, MD, MSc
Corresponding author, [theston@uw.edu](mailto:theston@uw.edu)
On behalf of Daniel Y Tsybulnik and Justin J Gillette
