# Supplementary material for: Variability among large language models in assessing CONSORT compliance of published randomized clinical trials
Source: PLoS One. 2026 Sep 22;21(9):e0358873. doi: 10.1371/journal.pone.0358873 (PMC13596804; doi:10.1371/journal.pone.0358873)
Supplement: Peer review history

# Peer review: Decision Letter 0

**DOI:** 10.1371/journal.pone.0358873.r001

**Authors:** Farshid Danesh

4 Jun 2026

Dear Dr. Heston,

Thank you for submitting your manuscript to PLOS ONE. After careful consideration, we feel that it has merit but does not fully meet PLOS ONE’s publication criteria as it currently stands. Therefore, we invite you to submit a revised version of the manuscript that addresses the points raised during the review process.

Please submit your revised manuscript by Jul 19 2026 11:59PM. If you will need more time than this to complete your revisions, please reply to this message or contact the journal office at plosone@plos.org. When you're ready to submit your revision, log on to https://www.editorialmanager.com/pone/ and select the 'Submissions Needing Revision' folder to locate your manuscript file.

- A letter that responds to each point raised by the academic editor and reviewer(s). You should upload this letter as a separate file labeled 'Response to Reviewers'.
- A marked-up copy of your manuscript that highlights changes made to the original version. You should upload this as a separate file labeled 'Revised Manuscript with Track Changes'.
- An unmarked version of your revised paper without tracked changes. You should upload this as a separate file labeled 'Manuscript'.

If you would like to make changes to your financial disclosure, please include your updated statement in your cover letter. Guidelines for resubmitting your figure files are available below the reviewer comments at the end of this letter.

If applicable, we recommend that you deposit your laboratory protocols in protocols.io to enhance the reproducibility of your results. Protocols.io assigns your protocol its own identifier (DOI) so that it can be cited independently in the future. For instructions see: https://journals.plos.org/plosone/s/submission-guidelines#loc-laboratory-protocols. Additionally, PLOS ONE offers an option for publishing peer-reviewed Lab Protocol articles, which describe protocols hosted on protocols.io. Read more information on sharing protocols at https://plos.org/protocols?utm_medium=editorial-email&utm_source=authorletters&utm_campaign=protocols.

As the corresponding author, your ORCID iD is verified in the submission system and will appear in the published article. PLOS supports the use of ORCID, and we encourage all coauthors to register for an ORCID iD and use it as well. Please encourage your coauthors to verify their ORCID iD within the submission system before final acceptance, as unverified ORCID iDs will not appear in the published article. *Only*  the individual author can complete the verification step; PLOS staff *cannot*  verify ORCID iDs on behalf of authors.

We look forward to receiving your revised manuscript.

Kind regards,

Farshid Danesh, Ph.D.

Academic Editor

PLOS One

Journal Requirements:

When submitting your revision, we need you to address these additional requirements.

1. Please ensure that your manuscript meets PLOS ONE's style requirements, including those for file naming. The PLOS ONE style templates can be found at

https://journals.plos.org/plosone/s/file?id=wjVg/PLOSOne_formatting_sample_main_body.pdf and

https://journals.plos.org/plosone/s/file?id=ba62/PLOSOne_formatting_sample_title_authors_affiliations.pdf

2. Please remove your figures from within your manuscript file, leaving only the individual TIFF/EPS image files, uploaded separately. These will be automatically included in the reviewers’ PDF.

3. Please include captions for your Supporting Information files at the end of your manuscript, and update any in-text citations to match accordingly. Please see our Supporting Information guidelines for more information: http://journals.plos.org/plosone/s/supporting-information.

4. If the reviewer comments include a recommendation to cite specific previously published works, please review and evaluate these publications to determine whether they are relevant and should be cited. There is no requirement to cite these works unless the editor has indicated otherwise.

[Note: HTML markup is below. Please do not edit.]

Reviewers' comments:

Reviewer's Responses to Questions

**Comments to the Author**

1. Is the manuscript technically sound, and do the data support the conclusions?

Reviewer #1: Partly

Reviewer #2: Partly

**********

2. Has the statistical analysis been performed appropriately and rigorously?

Reviewer #1: No

Reviewer #2: No

**********

3. Have the authors made all data underlying the findings in their manuscript fully available?

The PLOS Data policy

Reviewer #1: Yes

Reviewer #2: Yes

**********

4. Is the manuscript presented in an intelligible fashion and written in standard English?

Reviewer #1: Yes

Reviewer #2: Yes

**********

Reviewer #1: The manuscript addresses a timely and relevant topic by examining the ability of large language models (LLMs) to evaluate CONSORT compliance in randomized controlled trials; however, several substantive issues across all sections limit the scientific rigor and interpretability of the work. The Introduction lacks a precise articulation of the methodological gap that the manuscript intends to address. While peer-review limitations and CONSORT guidelines are summarized, the authors do not anchor these points to a coherent rationale for conducting a comparative LLM evaluation.The Methods section, while describing the use of a 1 / 0.5 / 0 scoring framework, does not provide the operational details necessary to ensure reproducibility. The manuscript does not specify the exact prompts used for each model, the temperature and generation parameters, the format in which articles were provided to the models (PDF, plain text, or processed segments), or the criteria each LLM used to internally assign “fully met,” “partially met,” or “unmet” scores for each CONSORT item. Since each model scores its own outputs without a human reference standard, the methodological implications of relying solely on self-evaluation are not addressed. Additionally, the manuscript does not clarify whether multiple runs were performed to account for model variability, how potential hallucinations were mitigated, or whether scoring consistency was examined across repeated evaluations. These omissions limit the transparency and reproducibility of the analysis. Although the authors note that they included the first 20 PubMed search results for “immunology” and “randomized controlled trial,” the rationale for selecting a sequential “first-20” sample is not discussed. Because PubMed’s default ranking does not reflect methodological quality, trial characteristics, or field representativeness, the authors should clarify why this approach was considered appropriate for evaluating LLM performance. Alternative sampling strategies—such as random sampling, stratified selection, or screening based on predefined inclusion criteria—could offer a more representative dataset. Without such justification, the generalizability of the findings remains uncertain. The Results section presents overall compliance percentages clearly but lacks item-level granularity, variability measures beyond confidence intervals, and formal agreement metrics such as Cohen’s κ or ICC, which would meaningfully strengthen the findings. Moreover, the use of a 90% compliance threshold is arbitrary and insufficiently justified. In the Discussion, the authors reiterate general statements about peer review weaknesses but provide limited interpretation of why models performed differently or which CONSORT domains contributed most to variability. The section overstates the implications of the findings despite the modest sample size and absence of a direct human-expert comparison group. The limitations section does not adequately address important methodological constraints such as potential hallucination, prompt sensitivity, domain specificity, or lack of external validation.The manuscript compares three LLMs, yet the prompts provided to Claude differ from those given to the other models. This introduces a major methodological confound, as any observed differences may reflect prompt variability rather than inherent model performance. A valid comparative evaluation requires identical instructions, inputs, and task framing across all models. The absence of prompt standardization substantially limits the interpretability of the findings. The Conclusions section does not succinctly synthesize the study’s key quantitative findings and instead revisits conceptual commentary already presented in the Discussion; it should clearly highlight what the study discovered, rather than restate known literature. Finally, the reference list contains several concerning inconsistencies: multiple citations dated 2025 lack DOIs. Overall, the manuscript explores an important concept but requires substantial revision to improve methodological transparency, analytical depth, interpretive accuracy, and reference validity before it can be considered for publication.

Reviewer #2: Thank you for the opportunity to review this manuscript entitled “Large Language Models for Detecting CONSORT Guideline Compliance in Published Randomized Clinical Trials: A Cross-Sectional Evaluation Study.” The manuscript addresses a timely and relevant topic: the potential use of large language models to support assessment of reporting guideline compliance in randomized controlled trials. This is an important issue for peer review, editorial workflows, research transparency, and clinical trial reporting quality.

The study evaluates 20 randomized controlled trials published between 2015 and 2024 in immunology journals and compares the assessments of three LLMs—ChatGPT-4o, Gemini 2.5 Pro, and Claude Sonnet 4, across 37 CONSORT 2010 subpoints. The reported mean compliance scores differed substantially across models: 81% for ChatGPT-4o, 68% for Claude Sonnet 4, and 55% for Gemini 2.5 Pro. The study therefore provides potentially useful evidence that LLM-based assessments of CONSORT compliance are highly model-dependent.

However, I have several major concerns that should be addressed before the manuscript can be considered technically sound.

First, the central limitation is the absence of an expert human reference standard. The manuscript repeatedly refers to LLM “accuracy” and states that the observed overall compliance rate validates model accuracy. However, no independent assessment by CONSORT experts, clinical trial methodologists, or trained human reviewers was conducted. Agreement with previously reported average compliance rates is not sufficient to validate accuracy at the article level or item level. At most, the current design supports conclusions about differences among LLM scoring patterns, not about the true correctness of those scores.

Second, the conclusions overstate the evidence. The finding that the overall LLM-assessed compliance rate is similar to previously reported rates does not prove that the models accurately identified reporting deficiencies. Similar aggregate averages may occur even if individual item-level classifications are frequently wrong. The authors should substantially revise the language throughout the manuscript, especially in the Abstract, Discussion, and Conclusions. Terms such as “accuracy,” “validated,” and “performance” should be replaced or qualified unless a human gold-standard comparison is added.

Third, the sampling strategy is insufficiently described and may introduce bias. The Methods section states that the first 20 trials meeting inclusion criteria were selected from PubMed using the search strategy “randomized controlled trial [Publication Type] AND immunology.” The manuscript should specify the date of search, sorting method, screening process, inclusion and exclusion criteria, journal list, and reasons for exclusion. Selecting the “first 20” eligible studies is not clearly reproducible and may not yield a representative sample.

Fourth, the prompt design appears to differ across models. ChatGPT-4o and Gemini 2.5 Pro reportedly received standardized detailed prompts, while Claude Sonnet 4 was evaluated using a simplified binary assessment prompt. This creates a major confounding factor. If the purpose is to compare models, the input, prompt structure, scoring instructions, and output format should be standardized across all models. If the purpose is to test prompt complexity, this should be designed explicitly as a factorial comparison in which all models are tested under comparable prompt conditions.

**********

what does this mean?). If published, this will include your full peer review and any attached files.

If you choose “no”, your identity will remain anonymous but your review may still be made public.

**Do you want your identity to be public for this peer review?**  For information about this choice, including consent withdrawal, please see our Privacy Policy

Reviewer #1: No

Reviewer #2: **Yes:** Rasoul Zavaraqi

**********

[NOTE: If reviewer comments were submitted as an attachment file, they will be attached to this email and accessible via the submission site. Please log into your account, locate the manuscript record, and check for the action link "View Attachments". If this link does not appear, there are no attachment files.]

To ensure your figures meet our technical requirements, please review our figure guidelines: https://journals.plos.org/plosone/s/figures

You may also use PLOS’s free figure tool, NAAS, to help you prepare publication quality figures: https://journals.plos.org/plosone/s/figures#loc-tools-for-figure-preparation.

NAAS will assess whether your figures meet our technical requirements by comparing each figure against our figure specifications.

# Author comment: Author response to Decision Letter 1

**DOI:** 10.1371/journal.pone.0358873.r002

14 Jul 2026

Our full point-by-point responses to all reviewer and editor comments are provided in the uploaded "Response to Reviewers" file.

Attachment Submitted filename: Response_to_Reviewers.docx

# Peer review: Decision Letter 1

**DOI:** 10.1371/journal.pone.0358873.r003

**Authors:** Farshid Danesh

28 Aug 2026

Dear Dr. Heston,

Thank you for submitting your manuscript to PLOS One. After careful consideration, we feel that it has merit but does not fully meet PLOS One’s publication criteria as it currently stands. Therefore, we invite you to submit a revised version of the manuscript that addresses the points raised during the review process.

Please submit your revised manuscript by Oct 12 2026 11:59PM. If you will need more time than this to complete your revisions, please reply to this message or contact the journal office at plosone@plos.org. When you're ready to submit your revision, log on to https://www.editorialmanager.com/pone/ and select the 'Submissions Needing Revision' folder to locate your manuscript file.

- A letter that responds to each point raised by the academic editor and reviewer(s). You should upload this letter as a separate file labeled 'Response to Reviewers'.
- A marked-up copy of your manuscript that highlights changes made to the original version. You should upload this as a separate file labeled 'Revised Manuscript with Track Changes'.
- An unmarked version of your revised paper without tracked changes. You should upload this as a separate file labeled 'Manuscript'.

If you would like to make changes to your financial disclosure, please include your updated statement in your cover letter. Guidelines for resubmitting your figure files are available below the reviewer comments at the end of this letter.

If applicable, we recommend that you deposit your laboratory protocols in protocols.io to enhance the reproducibility of your results. Protocols.io assigns your protocol its own identifier (DOI) so that it can be cited independently in the future. For instructions see: https://journals.plos.org/plosone/s/submission-guidelines#loc-laboratory-protocols. Additionally, PLOS One offers an option for publishing peer-reviewed Lab Protocol articles, which describe protocols hosted on protocols.io. Read more information on sharing protocols at https://plos.org/protocols?utm_medium=editorial-email&utm_source=authorletters&utm_campaign=protocols.

As the corresponding author, your ORCID iD is verified in the submission system and will appear in the published article. PLOS supports the use of ORCID, and we encourage all coauthors to register for an ORCID iD and use it as well. Please encourage your coauthors to verify their ORCID iD within the submission system before final acceptance, as unverified ORCID iDs will not appear in the published article. *Only*  the individual author can complete the verification step; PLOS staff *cannot*  verify ORCID iDs on behalf of authors.

We look forward to receiving your revised manuscript.

Kind regards,

Farshid Danesh, Ph.D.

Academic Editor

PLOS One

Journal Requirements:

If the reviewer comments include a recommendation to cite specific previously published works, please review and evaluate these publications to determine whether they are relevant and should be cited. There is no requirement to cite these works unless the editor has indicated otherwise.

Please review your reference list to ensure that it is complete and correct. If you have cited papers that have been retracted, please include the rationale for doing so in the manuscript text, or remove these references and replace them with relevant current references. Any changes to the reference list should be mentioned in the rebuttal letter that accompanies your revised manuscript. If you need to cite a retracted article, indicate the article’s retracted status in the References list and also include a citation and full reference for the retraction notice.

[Note: HTML markup is below. Please do not edit.]

Reviewers' comments:

Reviewer's Responses to Questions

**Comments to the Author**

Reviewer #2: All comments have been addressed

Reviewer #3: (No Response)

**********

2. Is the manuscript technically sound, and do the data support the conclusions?

Reviewer #2: Yes

Reviewer #3: Yes

**********

3. Has the statistical analysis been performed appropriately and rigorously?

Reviewer #2: Yes

Reviewer #3: Yes

**********

4. Have the authors made all data underlying the findings in their manuscript fully available?

The PLOS Data policy

Reviewer #2: Yes

Reviewer #3: Yes

**********

5. Is the manuscript presented in an intelligible fashion and written in standard English?

Reviewer #2: Yes

Reviewer #3: Yes

**********

Reviewer #2: The paper may be accepted. The authors have adequately addressed comments raised in a previous round of review and I feel that this manuscript is now acceptable for publication.

Reviewer #3: Reviewer Comments to the Author

I appreciate the opportunity to review this revised manuscript. The study addresses an interesting and timely topic regarding the use of large language models (LLMs) for evaluating adherence to CONSORT reporting standards in randomized clinical trials. The manuscript presents a relevant research question, a clearly described methodology, and an important discussion regarding the potential role and limitations of artificial intelligence-assisted manuscript evaluation.

Overall, the manuscript is well organized and the statistical approach is generally appropriate for the repeated-measures comparison of multiple LLMs. The authors have also provided a clear description of the evaluation framework and made the underlying data and analysis files available, which strengthens the transparency and reproducibility of the study.

However, I have several minor comments that may further improve the manuscript:

Clarification of methodological limitations:

Although the use of 20 randomized clinical trials provides a controlled comparison between models, the authors should further emphasize the limitations regarding sample size and generalizability. The selected articles represent a specific group of clinical trials, and the findings may not necessarily extend to other medical fields, study designs, or reporting contexts.

Statistical methodology clarification:

The statistical analysis appears appropriate; however, the manuscript would benefit from a clearer explanation of whether all assumptions underlying the repeated-measures ANOVA were evaluated and satisfied. Additional details regarding these assessments would improve methodological transparency.

Interpretation of model differences:

The manuscript demonstrates substantial variability among LLM assessments. The discussion could further explore possible reasons for these differences, including variations in model architecture, training data, prompt interpretation, or evaluation strategies.

Human reviewer comparison:

Since the manuscript discusses the potential application of LLMs in peer review, the authors may consider expanding the discussion regarding how LLM-based evaluations compare with expert human reviewers and whether future studies should include human-AI agreement analyses.

Language and presentation:

The manuscript is generally well written and understandable. Minor language editing may improve readability in some sections, particularly where sentences are lengthy or contain multiple concepts.

Overall, this is a valuable and relevant study that contributes to the emerging discussion of artificial intelligence applications in scientific evaluation. Addressing the above points would further strengthen the manuscript.

**********

what does this mean?). If published, this will include your full peer review and any attached files.

If you choose “no”, your identity will remain anonymous but your review may still be made public.

**Do you want your identity to be public for this peer review?**  For information about this choice, including consent withdrawal, please see our Privacy Policy

Reviewer #2: **Yes:** Rasoul Zavaraqi

Reviewer #3: No

**********

[NOTE: If reviewer comments were submitted as an attachment file, they will be attached to this email and accessible via the submission site. Please log into your account, locate the manuscript record, and check for the action link "View Attachments". If this link does not appear, there are no attachment files.]

To ensure your figures meet our technical requirements, please review our figure guidelines: https://journals.plos.org/plosone/s/figures

You may also use PLOS’s free figure tool, NAAS, to help you prepare publication quality figures: https://journals.plos.org/plosone/s/figures#loc-tools-for-figure-preparation.

NAAS will assess whether your figures meet our technical requirements by comparing each figure against our figure specifications.

Attachment Submitted filename: Reviewer Comments to the Author.pdf

# Author comment: Author response to Decision Letter 2

**DOI:** 10.1371/journal.pone.0358873.r004

4 Sep 2026

RESPONSE TO REVIEWERS PONE-D-25-55197R1.

Dear Dr. Danesh,

Thank you for your further review. All changes are marked in the tracked changes file.

Journal requirements. The reference list was verified. We corrected the formatting of references 2 and 25, updated reference 19 to the primary source, and added one reference (Moher 2001, now reference 20), shifting the subsequent numbering. Reference 17 is intentionally a retraction notice, cited and discussed as such.

Reviewer #2. We thank the reviewer for confirming the previous comments are resolved.

Reviewer #3

1. Sample size and generalizability. Both points were identified as limitations in the previous version and are now stated more explicitly in the Discussion limitations: a single specialty and narrow publication window may not extend to other fields, designs, or reporting contexts, and 20 trials support comparison between models but not generalization of any model's absolute score. The design remains matched to the question asked. Every model scored identical material, and the effect was large enough to resolve all three pairwise differences at n = 20.

2. ANOVA assumptions. We tested sphericity at the time of analysis and found it satisfied (Mauchly's W = 0.935, chi-square = 1.214 with 2 degrees of freedom, p = 0.545), so we used uncorrected degrees of freedom; the output was already in the deposited SPSS file but not reported in the text; it is now stated in the Statistical Analysis subsection and the Results.

3. Why the models differ. The Discussion now offers four candidate explanations: architecture, training data, strictness when a criterion is partially met, and prompt interpretation. These are anchored in the item-level results already reported, where divergence concentrated in judgment-dependent subpoints while structurally explicit subpoints showed near-complete agreement. The text states that the design cannot separate the four.

4. Human reviewer comparison. The previous version noted the absence of a human reference standard, and we maintain that model-to-model comparison answers a prerequisite question: if models disagree on the same text, at most one of them can track any fixed standard. The Discussion now grounds this in evidence: two trained reviewers scoring published trials against the CONSORT checklist agreed completely on explicit items but reached kappa of only 0.53 to 0.54 on judgment-dependent ones (Moher 2001), the same pattern seen between models here. Model-to-human agreement should therefore be judged against human-to-human agreement rather than treated as accuracy against truth. The recommendation for human-AI agreement studies now opens the future research paragraph.

5. Language. Seven long sentences were split, and one Discussion paragraph was divided. No numbers changed.

Sincerely,

Thomas F Heston, MD, MSc

Corresponding author, theston@uw.edu

On behalf of Daniel Y Tsybulnik and Justin J Gillette

Attachment Submitted filename: RESPONSE TO REVIEWERS.docx

# Editor report: Decision Letter 2

**DOI:** 10.1371/journal.pone.0358873.r005

**Authors:** Farshid Danesh

7 Sep 2026

Variability among large language models in assessing CONSORT compliance of published randomized clinical trials

PONE-D-25-55197R2

Dear Dr. Heston,

We’re pleased to inform you that your manuscript has been judged scientifically suitable for publication and will be formally accepted for publication once it meets all outstanding technical requirements.

Within one week, you’ll receive an e-mail detailing the required amendments. When these have been addressed, you’ll receive a formal acceptance letter and your manuscript will be scheduled for publication.

An invoice will be generated when your article is formally accepted. Please note, if your institution has a publishing partnership with PLOS and your article meets the relevant criteria, all or part of your publication costs will be covered. Please make sure your user information is up-to-date by logging into Editorial Manager at Editorial Manager® and clicking the ‘Update My Information' link at the top of the page. For questions related to billing, please contact billing support.

If your institution or institutions have a press office, please notify them about your upcoming paper to help maximize its impact. If they’ll be preparing press materials, please inform our press team as soon as possible -- no later than 48 hours after receiving the formal acceptance. Your manuscript will remain under strict press embargo until 2 pm Eastern Time on the date of publication. For more information, please contact onepress@plos.org.

Kind regards,

Farshid Danesh, Ph.D.

Academic Editor

PLOS One

Additional Editor Comments (optional):

Reviewers' comments:

# Editor report: Acceptance letter

**DOI:** 10.1371/journal.pone.0358873.r006

**Authors:** Farshid Danesh

PONE-D-25-55197R2

PLOS One

Dear Dr. Heston,

I'm pleased to inform you that your manuscript has been deemed suitable for publication in PLOS One. Congratulations! Your manuscript is now being handed over to our production team.

At this stage, our production department will prepare your paper for publication. This includes ensuring the following:

* All references, tables, and figures are properly cited

* All relevant supporting information is included in the manuscript submission,

* There are no issues that prevent the paper from being properly typeset

You will receive further instructions from the production team, including instructions on how to review your proof when it is ready. Please keep in mind that we are working through a large volume of accepted articles, so please give us a few days to review your paper and let you know the next and final steps.

Lastly, if your institution or institutions have a press office, please let them know about your upcoming paper now to help maximize its impact. If they'll be preparing press materials, please inform our press team within the next 48 hours. Your manuscript will remain under strict press embargo until 2 pm Eastern Time on the date of publication. For more information, please contact onepress@plos.org.

You will receive an invoice from PLOS for your publication fee after your manuscript has reached the completed accept phase. If you receive an email requesting payment before acceptance or for any other service, this may be a phishing scheme. Learn how to identify phishing emails and protect your accounts at https://explore.plos.org/phishing.

If we can help with anything else, please email us at customercare@plos.org.

Thank you for submitting your work to PLOS One and supporting open access.

Kind regards,

PLOS One Editorial Office Staff

on behalf of

Associate Professor Farshid Danesh

Academic Editor

PLOS One
